# Application of Augmented Reality in Robot-Assisted Mitral Valve Repair Surgery: A Feasibility Study

**DOI:** 10.1177/15569845251367418

**Published:** 2025-09-16

**Authors:** Jette J. Peek, Klaus Hildebrandt, Xucong Zhang, Rohit K. Kharbanda, Maurice A.P. Oudeman, Robert J.M. Klautz, Meindert Palmen, Edris A.F. Mahtab

**Affiliations:** 1 Department of Cardiothoracic Surgery, Erasmus University Medical Center, Rotterdam, The Netherlands; 2 Computer Graphics and Visualization Lab, TU Delft, The Netherlands; 3 Computer Vision Lab, TU Delft, The Netherlands; 4 Department of Cardiothoracic Surgery, Leiden University Medical Center, The Netherlands; 5 Instituut voor Hart en Long Chirurgie Nederland (IHLCN; Dutch Institute for Heart and Lung Surgery), Leiden, The Netherlands; 6 Department of Cardiothoracic Surgery, Amsterdam University Medical Center, The Netherlands

**Keywords:** augmented reality, robot assisted, mitral valve repair, image-guided surgery

## Abstract

**Objective:**

In mitral valve surgery, it is important to be aware of adjacent intraoperatively invisible anatomy, to avoid complications and enhance safety. In this feasibility study, we aimed to develop semi-automated intraoperative 3-dimensional (3D) augmented reality (3D-AR) overlays for robotic mitral valve repair.

**Methods:**

In 5 patients undergoing robot-assisted mitral valve repair, a 3D point cloud was generated, using intraoperatively recorded images from both eyes of the stereoscopic da Vinci camera (Intuitive Surgical, Sunnyvale, CA, USA). An intraoperative 3D-AR overlay was created using a scale-adaptive iterative closest point algorithm and landmarks placed on the mitral valve annulus. Finally, important anatomical structures such as the circumflex artery, Koch’s triangle, and aortic valve leaflets could be visualized as a 3D-AR overlay on top of the surgical vision. To evaluate the accuracy, these 3D point clouds were validated by calculating the 3D point cloud accuracy and landmark registration error (LRE).

**Results:**

The 3D point clouds and 3D-AR overlays were successfully created for all 5 patients. The 3D point clouds were accurate, with a median error of −0.92 mm, and the LRE was 5.12 mm. The time for creating the 3D-AR overlay was approximately 5 min. Besides creating the 3D-AR overlays, we could visualize the models directly within the robotic console during the surgical procedure.

**Conclusions:**

We present an algorithm for generating accurate semiautomatic 3D-AR overlays, visualizing essential anatomical structures during robot-assisted mitral valve repair. This may lead to automated intraoperative 3D-AR vision during robotic cardiac surgery, with the potential of increasing safety, accuracy, and efficiency.

## Graphical abstract

**Graphical Abstract:**
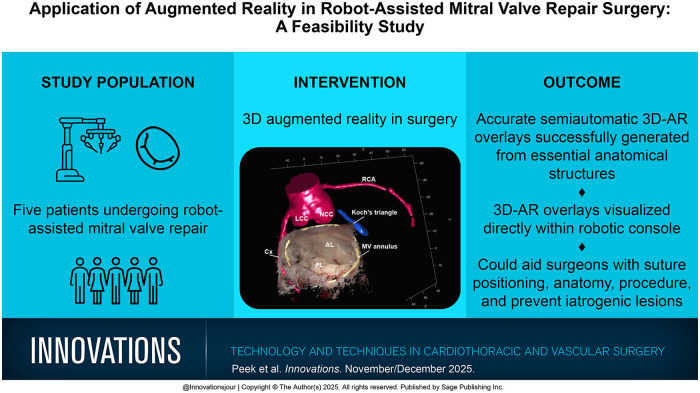

**Central Message**

A method was developed to generate precise, semiautomatic 3D-AR overlays for visualizing critical anatomical structures during robot-assisted mitral valve repair. This approach provides a framework for real-time intraoperative 3D-AR visualization in robotic cardiac surgery, with the potential to enhance safety, accuracy, and overall efficacy.

## Introduction

Minimally invasive approaches for mitral valve repair, including robot-assisted surgery, constitute valuable alternatives to conventional mitral valve surgery.^1^ Robotic mitral valve repair yields similar short-term and long-term results and complication rates compared with other conventional and minimally invasive approaches.^1^ In both approaches, successful mitral valve repair depends on excellent valve visualization, which enables adequate analysis and structured repair of the mitral valve. To minimize the risk of iatrogenic lesions to vicinal anatomic structures, the surgeon must be aware of the intraoperatively invisible underlying anatomy. Iatrogenic lesions induced by annular sutures can include coronary artery occlusion, aortic leaflet perforation, or atrioventricular conduction blocks.^2–4^ Although these iatrogenic lesions are rare, the consequences may be severe for the patient.^2–4^ Research across various disciplines has shown that both preoperative 3-dimensional (3D) modeling and immersive virtual reality simulation enable accurate pulmonary parenchymal resections, reduce surgical time, shorten postoperative hospital stay, and improve procedural metrics.^5,6^ However, the currently available 3D modeling software does not fully represent the intraoperative view during cardiac surgery, when the heart is cardioplegic and unloaded on cardiopulmonary bypass (CPB). In contrast, augmented reality (AR) overlays enhance the surgical view by directly integrating preoperative 3D models derived from a computed tomography (CT) scan, aiding in identifying structures and visualizing hidden structures.^7^ AR can be applied for intraoperative surgical guidance directly in the robotic console display or on the thoracoscopic monitor.^8^ In this study, we aim to demonstrate the feasibility of AR during robotic mitral valve repair by creating a 3D-AR overlay of preoperative 3D models of the patient-specific anatomy onto (3D stereoscopic) robotic videos and images.

## Methods

**Fig. 1.**
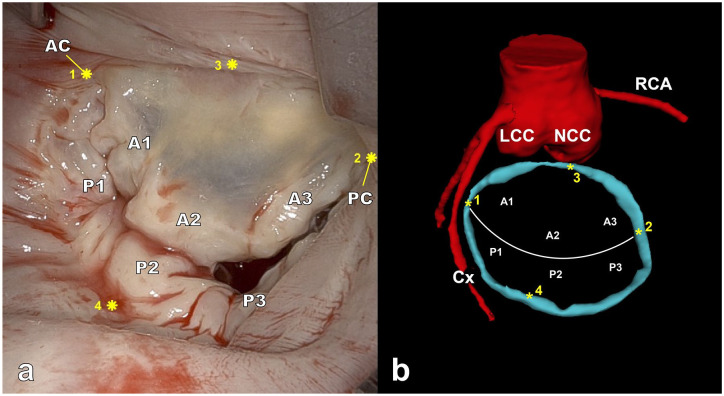
(a) Intraoperative view of the MV, showing 4 selected landmarks (marked with *) used for registration, (b) preoperative 3-dimensional model of the MV annulus (blue), with the corresponding 4 selected landmarks, aortic root, and coronary arteries (red). Landmarks 1 and 2 are placed at the anterior and posterior commissure, respectively. AC, anterior commissure; Cx, circumflex artery; LCC, left coronary cusp; MV, mitral valve; NCC, noncoronary cusp; PC, posterior commissure; RCA, right coronary artery.

In this prospective observational pilot study, we included 5 patients who underwent robot-assisted mitral valve repair surgery at Leiden University Medical Center. The study was approved by the local scientific committee, and all patients provided written informed consent. All patients suffered from severe primary mitral regurgitation and had a class I recommendation to undergo surgical mitral valve repair according to the current guidelines.^9^ Patients were selected for robotic mitral valve repair based on our local protocol, taking both valve-related and patient-related characteristics into account. During surgical workup, all patients received a contrast-enhanced thoracoabdominal CT scan for assessing intrathoracic proportions and peripheral vascular access for CPB cannulation. All patients underwent robotic mitral valve repair using the da Vinci X robotic platform (Intuitive Surgical, Sunnyvale, CA, USA). A stepwise workflow for applying landmark registration (i.e., aligning) for creating 3D-AR overlays was applied as described and shown in a schematic figure previously but with an added scaling component.^10^ Preoperatively, stereoscopic camera calibration of the camera (da Vinci 30° 3DHD endoscope) was performed to acquire intrinsic camera parameters.^11^ The structures of interest, circumflex artery (Cx), mitral valve annulus and leaflets, aortic valve cusps, and Koch’s triangle were segmented from the CT scan using 3D Slicer 5.6.0.^12^ Intraoperatively, stereoscopic images were acquired simultaneously as separate left and right eye video streams using the Blackmagic Multiview 4 (Blackmagic Design, Port Melbourne, Australia) and Elgato Cam Link 4K (Corsair, Milpitas, CA, USA). Subsequently, these images were undistorted and rectified applying the intrinsic camera parameters in the OpenCV package in Python 3.9.12.^13^ The undistorted images were imported in MATLAB R2023b (The MathWorks Inc, Natick, MA, USA) for point cloud creation and registration. A disparity map was generated using the undistorted images, and a 3D point cloud was created by computing the real-world 3D coordinates and depth of each pixel of the disparity map. This resulted in an intraoperative 3D point cloud (or reconstruction) of the surgical scene. Next, initial registration (i.e., alignment) of the 3D segmentation onto the 3D point cloud was performed using 4 to 8 3D key points located on the annulus (Fig. 1). Scale-adaptive iterative closest point was then applied to the point pairs to compute the optimal transformation matrix, which incorporates scaling, rotation, and translation,^14^ and is used to align the preoperative 3D model with the intraoperative 3D point cloud. Finally, both the 3D model and intraoperative 3D point cloud were directly displayed as a 3D-AR overlay in the robotic console using the TilePro application. As this was a feasibility study, the 3D-AR overlay was shown only after suture and ring placement to avoid influencing surgical decision-making during annular suture placement. To assess the feasibility, the 3D point clouds were validated by measuring the intraoperative diameter of the mitral valve on the 3D point clouds and comparing it with the implanted annuloplasty ring size. The landmark registration error was calculated between the intraoperative and preoperative landmark point sets and displayed as root mean squared distance (mm). An overlay was considered unsuccessful if the 3D model was visibly misaligned from the intraoperative visible mitral valve annulus.

## Results

**Fig. 2.**
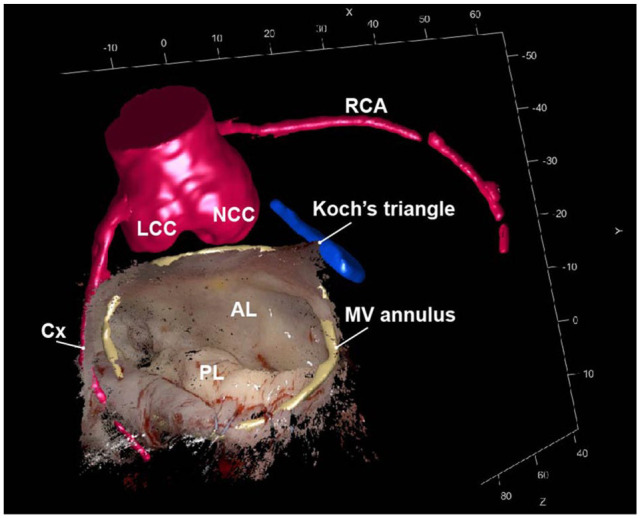
Intraoperative 3D point cloud together with AR overlay of the preoperative 3D segmentations. The close relationship between the MV annulus (yellow), aortic valve (red), and Koch’s triangle (blue) is clearly demonstrated. 3D, 3-dimensional; AL, anterior leaflet; Cx, circumflex artery; LCC, left coronary cusp; MV, mitral valve; NCC, noncoronary cusp; PL, posterior leaflet; RCA, right coronary artery.

All patients underwent successful robotic mitral valve repair. In all patients, segmental leaflet prolapse was corrected by implanting polytetrafluoroethylene neochords, and all patients underwent full ring annuloplasty (Physio II, Edwards Lifesciences, Irvine, CA, USA) to obtain annular remodeling (Table 1). The overall mean reprojection error of the stereoscopic camera calibration was 0.22 pixels. For all patients, a disparity map and 3D point cloud surface of the recorded mitral valves were created successfully. The median absolute error between the annuloplasty ring size and the measured 3D point cloud diameter was −0.92 mm (interquartile range [IQR], –3.57 to 2.44). Subsequently, 3D-AR overlays of the Cx artery, mitral valve annulus, Koch’s triangle, and aortic valve were created (Fig. 2, Supplemental Video 1). The median landmark registration error was 5.12 mm (IQR, 4.17 to 6.33). To avoid visual distractions, the view could be cropped, visualizing only the region of interest, and the 3D model visibility could be toggled individually for each anatomical structure. Using this 3D-AR overlay technique, surgeons were able to see what was situated underneath the intraoperative visible surface. In the future this could possibly aid less experienced surgeons in positioning the sutures near critical underlying anatomy, helping to prevent iatrogenic lesions and getting familiar with the procedure. The creation of these overlays took approximately 5 min; therefore, it was possible to run the algorithm intraoperatively and show the 3D-AR overlay with a slight delay in the robotic console via the TilePro functionality (Supplemental Video 1).

**Table 1.** Baseline Characteristics and MV Measurements for Included Patient Cases.

	Patient 1	Patient 2	Patient 3	Patient 4	Patient 5
Baseline characteristics					
Age, y	71	64	55	58	54
Sex	M	M	M	F	M
Coronary dominance	R	R	R	R	R
Valve pathology	Degenerative-FED; prolapse PMVL	Degenerative-FED; prolapse PMVL	Degenerative-Barlow; bileaflet prolapse	Degenerative-CTD; prolapse PMVL	Degenerative-Barlow; bileaflet prolapse
Repair strategy	Annuloplasty + neochorda P2	Annuloplasty + neochorda P2	Annuloplasty + neochorda P1, P2, P3	Annuloplasty + neochorda P2, P3	Annuloplasty + neochorda P2, P3
Valve measurements					
Preoperative MV annulus diameter, mm	38.9	53.46	59.08	50.84	43.1
Intraoperative MV annulus diameter, mm	29.81	37.08	42.88	33.13	31.05
AMVL length, mm	23	29	31	20	27
Annulus ring size	32	38	40	32	36
Rigid registration					
Scaling factor	0.72	0.71	0.61	0.68	0.79
Landmarks, *n*	8	5	6	4	4
LRE, mm	5.12	6.71	4.66	3.68	5.94
CT information					
Scan protocol	CTA thorax/abdomen	CTA thorax/abdomen	CTA TAVI	CTA thorax/abdomen	CT heart ECG-triggered
Slice thickness, mm	0.75	0.75	0.9	0.5	0.5
Slices, *n*	890	777	813	2,126	320

Abbreviations: AMVL, anterior mitral valve leaflet; CT, computed tomography; CTA, computed tomography angiography; CTD, connective tissue disorder; ECG, electrocardiography; F, female; FED, fibroelastic deficiency; LRE, landmark registration error; M, male; MV, mitral valve; P1, anterior or medial mitral valve scallop; P2, middle mitral valve scallop; P3, posterior or lateral scallop; PMVL, posterior mitral valve leaflet; R, right; TAVI, transcatheter aortic valve implantation.

## Discussion

In this pilot study, we demonstrated the feasibility of applying a 3D point cloud registration method for robotic mitral valve repair, as described previously for pulmonary surgery.^10^ To our knowledge, the application of such 3D-AR visualizations in robotic mitral valve surgery has not been previously described in the literature. To improve the mitral annulus registration and to account for the size difference between the intraoperative unloaded heart on CPB and the preoperative CT scan, we incorporated scaling into the algorithm. Without adding scaling, the 3D-AR overlay showed a significantly larger mitral valve annulus compared with the actual intraoperative diameter. The intraoperative 3D point clouds demonstrated high accuracy, as the median error was submillimetric between the 3D point cloud and annuloplasty ring measurements. The created 3D-AR overlays can aid the surgeons in identifying and actively avoiding the visualized neighboring structures that are invisible during surgery. This could potentially aid in enhancing precision and improving the safety of robotic mitral valve repair.

Several limitations and software improvements need to be mentioned. Currently, automated real-time 3D-AR overlays cannot be created as landmark selection for registration is performed manually. Therefore, the AR overlay could be displayed only in the TilePro application with a slight delay. Automation of the registration is a prerequisite for real-time visualization, together with the creation of a graphical user interface combining the software packages currently used. Artificial intelligence in combination with real-time point cloud generation could facilitate this process. However, continuous real-time 3D-AR overlay may not be necessary as it is primarily needed at specific moments during the surgery,^15^ especially during suture placement for ring annuloplasty.

Another concern is the use of rigid registration to create the 3D-AR overlays, as the anatomy of the heart on the CT scan differs from the intraoperative arrested and void situation. Nevertheless, rigid registration seemed accurate and feasible for the 5 cases in this study. Nonrigid registration techniques could be considered in future work, to better account for the intraoperative anatomical changes of the Cx and the mitral annulus. This might improve the registration accuracy and LRE by also including the intraoperative deformations of the left atrium to the registration of the overlay.

Furthermore, contrast CT scans were used as input for the AR overlay, which are ideally triggered by electrocardiography for optimal coronary artery visualization, whereas 3D echocardiography is better applicable for visualization of the mitral valve and is readily available in the operating room. Exploring methods that incorporate 3D echocardiography as an input for the 3D-AR overlays may result in adequate localization of mitral annular calcifications, guiding decalcification, and may be used in selecting the desired annular ring size for future applications.^15^

Besides technical development, validation is essential for clinical implementation. Future work should address validity and accuracy. Validation should also be performed for patients with a left dominant coronary system, as the risk of injuries might be higher in that population. Usability or other validation assessments were not yet incorporated in this feasibility study. However, the optimal method for evaluating the accuracy and validity of surgical AR systems is still researched.^8^

## Conclusions

This study presents an algorithm transforming the robot vision into a 3D point cloud surface, enabling registration and visualization of 3D models as AR overlays. This could potentially aid surgeons in avoiding iatrogenic lesions during robotic mitral valve repair. This method can be the framework of automatically intraoperative AR vision during robotic cardiac surgery. Validation to ensure that the procedure becomes safer and more accurate with this 3D-AR–guided surgical workflow remains crucial for clinical application in robotic mitral valve repair.
